# RNA-Seq-based investigation of interferon-mediated antiviral activity of chlorophyll a from Tetraselmis sp.

**DOI:** 10.17179/excli2025-8841

**Published:** 2026-01-09

**Authors:** Nalae Kang, Eun-A Kim, Yeon-Ji Lee, Seong-Yeong Heo, Jun-Ho Heo, Won-Kyu Lee, Yong-Kyun Ryu, Taeho Kim, Soo-Jin Heo

**Affiliations:** 1 Jeju Bio Research Center, Korea Institute of Ocean Science and Technology (KIOST), Jeju 63349, Republic of Korea; 2 Department of Marine Technology & Convergence Engineering, University of Science and Technology (UST), Daejeon 34113, Republic of Korea

**Keywords:** Chlorophyll a, Tetraselmis sp., antiviral activity, RNA-seq, IFITs

## Abstract

*Tetraselmis* sp., a marine microalga amenable to mass cultivation, possesses antiviral properties, partly attributed to chlorophyll a. However, the underlying antiviral mechanisms remain poorly characterized. In this study, we investigated the antiviral activity and mode of action of chlorophyll a derived from *Tetraselmis* sp. by performing transcriptomic analyses on three experimental groups: untreated, uninfected cells; Zika virus (ZIKV)-infected cells; and chlorophyll a-treated, ZIKV-infected cells. Treatment with 5 µM chlorophyll a induced differential expression of genes associated with interferon-inducible antiviral responses. Gene ontology analysis revealed significant enrichment of biological processes such as “response to external stimulus” and “response to biotic stimulus.” Notably, Venn diagram analysis of 130 differentially expressed genes (DEGs) demonstrated restoration of key interferon-stimulated genes, including interferon-induced protein with tetratricopeptide repeats (IFIT)1, IFIT2, IFIT3, and IFIT5, which was further validated by quantitative PCR and immunocytochemistry. These findings suggest that chlorophyll a from *Tetraselmis* sp. exerts antiviral effects primarily through modulation of interferon-mediated pathways.

See also the graphical abstract(Fig. 1).

## Introduction

Zika virus (ZIKV) is primarily transmitted between humans and non-human primates by *Aedes* mosquitoes (*Aedes aegypti *and *Aedes albopictus*) (Sadeghieh et al., 2021[27]). ZIKV infection has been linked to serious complications, including congenital microcephaly, Guillain-Barré syndrome, and meningoencephalitis, with neurological manifestations being particularly concerning due to their association with severe birth defects and long-term health consequences (Chen et al., 2020[3]).

Climate change has been shown to increase the risk of mosquito-borne epidemics by expanding mosquito habitats through shifts in temperature, precipitation, and humidity. These environmental changes extend mosquito activity periods and enhance their reproductive cycles, thereby increasing the transmission potential of arboviruses such as ZIKV (Barrera et al., 2011[2]; Paz and Semenza, 2016[21]; Van Wyk et al., 2023[32]; Wu et al., 2007[35]). During the 2016 ZIKV outbreak in Brazil, regions experiencing El Niño conditions, characterized by elevated temperatures and severe flooding, coincided with areas of high ZIKV prevalence (Paz and Semenza, 2016[21]), further suggesting that extreme climatic events contribute to the spread of *Aedes* mosquitoes and consequently, ZIKV (Yang and Sarfaty, 2016[40]). Despite this growing threat, no approved antiviral therapies or ingredients are currently available to treat ZIKV infection.

*Tetraselmis* sp., a marine microalga of the division *Chlorophyta*, is characterized by its green pigmentation, unicellular structure, and spherical to slightly flattened morphology. This genus is recognized for its high protein, fatty acid, carotenoid, and chlorophyll content (Paterson et al., 2023[20]), the levels of which vary depending on cultivation conditions, particularly salinity and nitrogen availability. Notably, *Tetraselmis* sp. can be cultivated year-round in semi-open raceway systems (Lee et al., 2021[16]), and its chlorophyll derivatives exhibit scalability and environmental sustainability, making them suitable for industrial-scale applications.

Chlorophyll derivatives, particularly chlorophyll *a* (CA), are lipophilic pigments essential for photosynthesis and vary in abundance based on the organism's photosynthetic capacity (Farahin et al., 2021[8]; Gao et al., 2024[9]; Yao et al., 2015[42]). In addition to their biological functions, chlorophyll derivatives have been shown to confer multiple health benefits, including anticancer, antimutagenic, antioxidant, radioprotective, antimicrobial, and anti-inflammatory effects (Hanafy et al., 2021[10]; Pucci et al., 2021[22]; Romodin and Lysenko, 2022[25]; Sun et al., 2024[29]). Our previous study demonstrated that CA isolated from *Tetraselmis* sp. exhibited antiviral activity against ZIKV, as evidenced by reductions in infectious viral titers and NS1 mRNA levels in infected cells. Additionally, the molecular docking simulations predicted interactions between CA and ZIKV (Kang et al., 2024[13]). However, the molecular mechanisms underlying this antiviral effect remain unclear.

RNA sequencing (RNA-Seq) is a powerful high-throughput technology for identifying functional food ingredients and drug candidates and elucidating their mechanisms of action through comprehensive transcriptomic and genomic analyses (Chen et al., 2021[4]; Hu et al., 2023[11]; Zhang et al., 2021[45]). Several recent studies have used RNA-Seq to profile gene expression changes in ingredient-treated cells, offering insights into therapeutic mechanisms (Jiang et al., 2022[12]; Kuang et al., 2022[14]; Li et al., 2024[18]; Xiang et al., 2017[37]; Zhang et al., 2021[45]).

In this study, we aimed to investigate the antiviral activity and mode of action of CA derived from *Tetraselmis* sp. by analyzing the transcriptomic profiles of CA-treated, ZIKV-infected cells through RNA-Seq to confirm the potential of CA derived from *Tetraselmis* sp. in anti-ZIKV therapies.

## Materials and Methods

### Preparation of CA from Tetraselmis sp.

**Figure 2:**
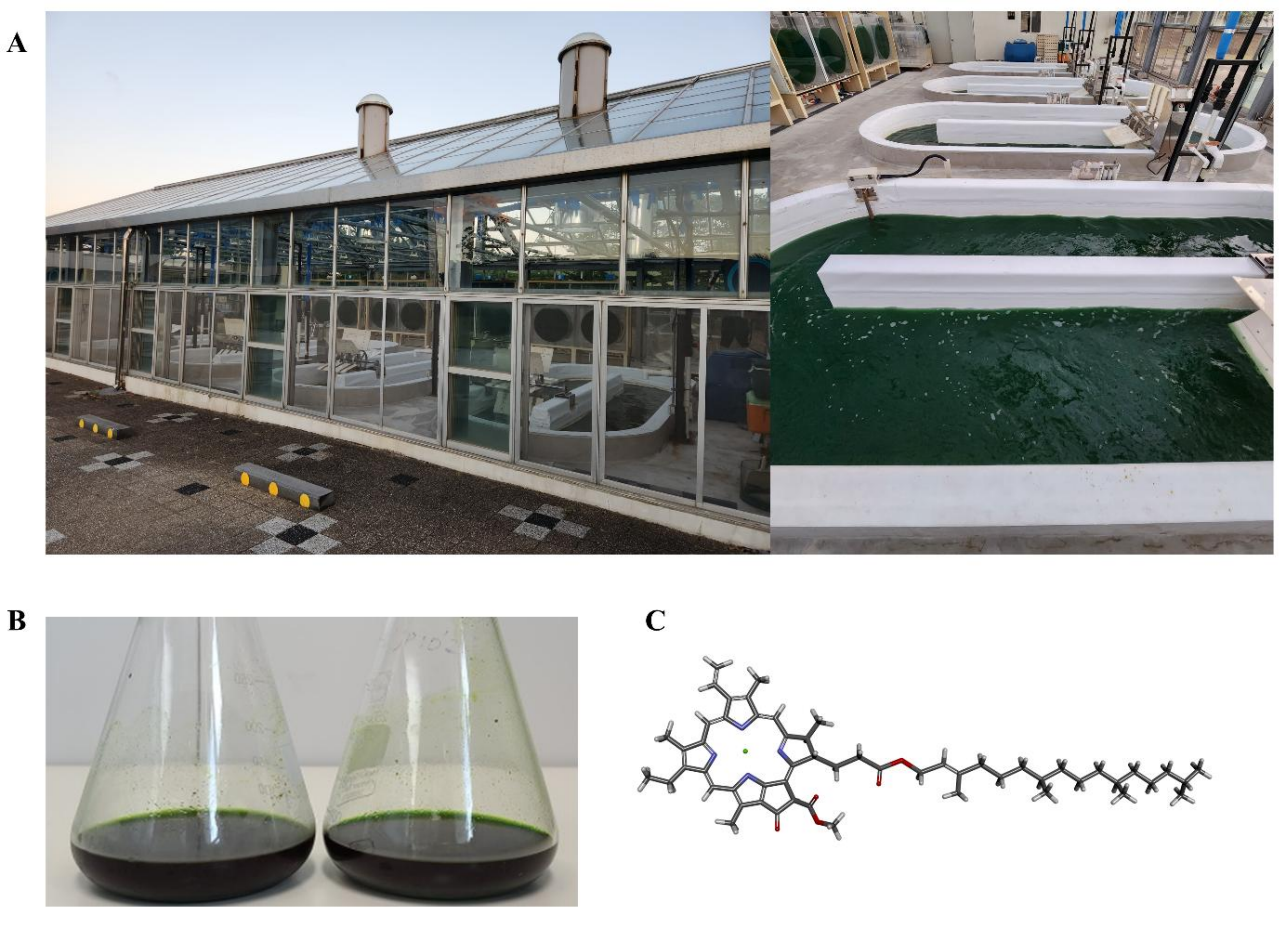
Chlorophyll *a* (CA) derived from *Tetraselmis* sp. (A) Mass production system for *Tetraselmis* sp. (B) Acetone (90 %) extracts of *Tetraselmis* sp. (C) Chemical structure of CA

*Tetraselmis* sp. was mass-cultivated in a semi-open raceway system, as previously described (Kang et al., 2024[13]; Lee et al., 2021[16]). The system has been meticulously engineered in conjunction with a greenhouse, thereby ensuring the sustainable cultivation of marine microalgae irrespective of external conditions (Figure 2A(Fig. 2)). The harvested biomass was lyophilized and stored at −80 °C until use. CA was extracted through a series of steps, including 90 % acetone extraction (Figure 2B(Fig. 2)), filtration using 1 µm filter paper, vacuum evaporation, and purified via a Media Flash C18 column, a Porasil silica column, and a Shimpack C18 column, following established protocols (Kang et al., 2024[13]). The CA structure was characterized by using liquid chromatography-mass spectrometry (LC-MS) and nuclear magnetic resonance (NMR) analyses (Figure 2C(Fig. 2)).

### Cell line and virus

Vero E6 cells were obtained from the American Type Culture Collection (Manassas, VA, USA) and maintained in Dulbecco's Modified Eagle Medium (DMEM; Gibco, Waltham, MA, USA) supplemented with 10 % fetal bovine serum (FBS; Gibco), 1 % 10 mM Hydroxyethyl piperazine ethane sulfonic acid (HEPES; Thermo Fisher Scientific, Waltham, MA, USA), and 1 % antibiotic-antimycotic solution (Thermo Fisher Scientific). Cells were incubated at 37 °C in a humidified atmosphere containing 5 % CO₂. ZIKV was obtained from the Korea Centers for Disease Control and Prevention (KCDC, Cheongju, South Korea). Virus stocks (1 × 10⁵ PFU/mL) were titrated by plaque assay and stored at −80 °C.

### Cytotoxicity assay

Vero E6 cells (2 × 10⁴ cells/well, in 96-well plates) were subjected to an initial incubation period of 16 h, followed by treatment with various concentrations of CA for an additional 72 h. Cell viability was evaluated using the WST-1 assay (DoGen, Seoul, South Korea). 

### Sample treatment and virus infection

Vero E6 cells (1 × 10⁶ cells/well, in 6-well plates) were incubated in DMEM containing 10 % FBS, 1 % HEPES and 1 % antibiotic-antimycotic solution until confluency was reached. Cells were washed with 1 mL Dulbecco's Phosphate Buffered Saline (DPBS) and applied with CA (2.5, 5, 10, or 20 µM) with 2 mL of DMEM supplemented with 2 % FBS, 1 % HEPES and 1 % antibiotic-antimycotic solution for 2 h prior to infection with ZIKV at 0.01 multiplicity of infection (MOI). Untreated, uninfected cells served as the mock control, while virus-infected but untreated cells served as the positive control (PC). CA-treated, virus-infected cells comprised the experimental groups. After 48 h of incubation, cell culture supernatants were collected for plaque assays, and cells were harvested for RNA-Seq and quantitative PCR (qPCR) analyses to evaluate ZIKV-related gene expression. 

### Plaque assay

Plaque assays were performed as described previously (Kang et al., 2024[13]). Vero E6 cells (1 × 10⁶ cells/well, in 6-well plates) were incubated in DMEM containing 10 % FBS, 1 % HEPES, and 1 % antibiotic-antimycotic solution until confluency was reached. Cell culture supernatants collected for plaque assays as described in the section entitled “Sample treatment and virus infection” were serially diluted 10-fold in DMEM. The prepared cells were washed twice with DPBS, followed by the addition of 0.2 mL DMEM and 0.5 mL of each cell culture supernatant dilution per well. Following a 2 h adsorption period with gentle agitation every 15 min, the inoculum was removed, and 3 mL of DMEM/F12 containing 2 % Oxoid agarose was overlaid onto the cells. The cells were incubated at 37 °C with 5 % CO₂ for 5 d. The cells were fixed with 4 % formaldehyde for 1 h and subsequently stained with 0.1 % crystal violet for 30 min. Viral titers were calculated as follows: PFU/mL = Number of plaques / (dilution factor × volume of inoculum per well). 

### RNA-seq analysis

RNA-seq was conducted using total RNA extracted from three experimental groups of cells. Total RNA from Vero E6 cells was extracted using the acid guanidinium thiocyanate-phenol-chloroform method (Chomczynski and Sacchi, 1987[6]). For library preparation, the TruSeq Stranded Total RNA with Ribo-Zero H/M/R Gold kit (Illumina, San Diego, CA, USA) was used to deplete ribosomal RNA and generate sequencing-ready libraries. Sequencing was performed on an Illumina platform to obtain paired-end reads (101 bp). Raw reads underwent quality control and adapter trimming to remove low-quality bases and residual adapter sequences, yielding high-quality trimmed reads (Supplementary information, Figure S1-S2). These reads were mapped to the *Chlorocebus sabaeus* reference genome (NCBI Assembly: GCF_015252025.1) using the HISAT2 program. Alignment files were processed with StringTie to assemble transcripts and quantify gene expression. Transcript abundance was calculated as raw read count, fragments per kilobase of transcript per million mapped reads (FPKM), and transcripts per million (TPM). Differential gene expression analysis was performed using read counts, FPKM, and TPM values in the DESeq2 package. Genes with an absolute fold change ≥ 2 and raw *p* value < 0.05 (based on the *nbinomWaldTest*) were considered significantly differentially expressed. Functional classification and gene annotation of differentially expressed genes (DEGs) were carried out using g:Profiler based on gene ontology (GO) and pathway information. 

### qPCR assay

Total RNA from Vero E6 cells was extracted using the acid guanidinium thiocyanate-phenol-chloroform method (Chomczynski and Sacchi, 1987). RNA was reverse-transcribed into complementary DNA (cDNA) using the cDNA synthesis kit (Applied Biosystems, Waltham, MA, USA). qPCR was performed using the SYBR green method on a QuantStudio 3 (Thermo Fisher Scientific) with the cycling conditions as follows: 95 °C for 10 min, followed by 40 cycles of 95 °C for 15 s and 60 °C for 1 min. Gene expression levels were calculated using the crossing point method. Primer sequences are listed in Table 1(Tab. 1). 

### Immunocytochemistry

Vero E6 cells (1 × 10^5^ cells/well, in 8-well cell culture slides) were incubated in DMEM containing 10 % FBS, 1 % HEPES and 1 % antibiotic-antimycotic solution until confluency was reached. Cells were washed with 0.2 mL DPBS and applied with CA (5 µM) with 0.2 mL of DMEM supplemented with 2 % FBS, 1 % HEPES and 1 % antibiotic-antimycotic solution for 2 h prior to infection with ZIKV at 0.01 MOI. Untreated, uninfected cells served as the mock control, while virus-infected but untreated cells served as the positive control (PC). CA-treated, virus-infected cells comprised the experimental groups. After 48 h of incubation, protein expression levels of cells were measured by immunocytochemistry assay. Cells were washed with 0.2 mL DPBS 3 times, and fixed with 4 % paraformaldehyde for 10 min at 37 °C. Cells were washed with 0.2 mL DPBS 3 times again, and permeabilized with 0.2 mL 0.1 % Triton X-100 in 1X DPBS, and incubated for 15 min at 25 °C. Then, cells were washed with 0.2 mL DPBS 3 times, and blocked with 0.25 mL 2 % BSA in 1X DPBS, and incubated for 60 min at 25 °C. After that, cells were immunostained with primary antibody (IFIT2 and IFIT3, Thermo Fisher Scientific), secondary antibody (Alexa Fluor^TM^ 488, Thermo Fisher Scientific), and hoechst33342 (Invitrogen, Waltham, MA, USA) sequentially. Cell morphology was examined using a Leica THUNDER 3D DMi8 fluorescence microscope (Leica Microsystems, Wetzlar, Germany), and positively stained areas were quantified using ImageJ software.

### Statistical analysis

All in vitro experimental data are presented as the mean ± standard deviation from three independent experiments. Statistical analyses were conducted using GraphPad Prism version 10 (GraphPad Software, San Diego, CA, USA). One-way ANOVA followed by Dunnett's multiple comparison test was used to assess statistical significance. Differences were considered statistically significant at *p* < 0.05.

## Results

### Inhibitory effects of CA on ZIKV infection

**Figure 3:**
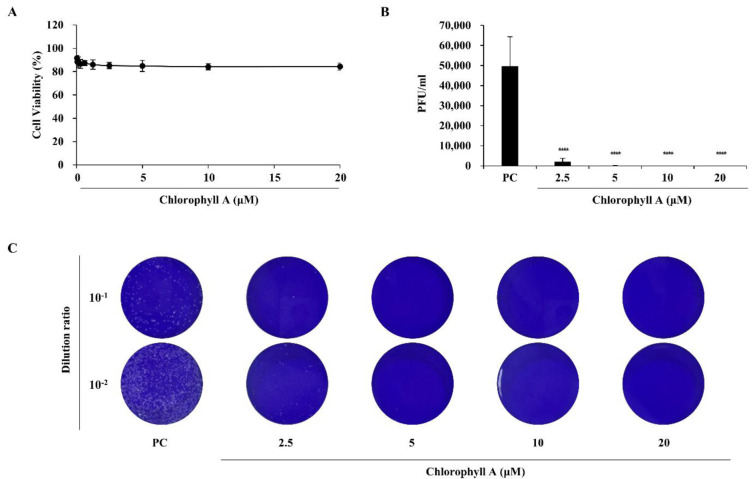
Inhibitory effects of CA on Zika virus (ZIKV) infection. (A) Cytotoxicity of CA in Vero E6 cells. (B) Plaque titers and (C) representative plaque assay images showing the antiviral activity of CA

CA was extracted from mass-cultivated *Tetraselmis* sp. using 90 % acetone and subsequently purified, and characterized as a main compound using LC-MS and NMR analyses as described previously (Figure 2(Fig. 2); Kang et al., 2024[13]). The antiviral effect of CA against ZIKV was evaluated by measuring the rate of viral plaque formation (Figure 3(Fig. 3)), with ZIKV-infected cells serving as the PC. CA treatment (2.5, 5, 10, and 20 µM) significantly reduced infectious viral particle levels without inducing cytotoxicity (Figure 3A-C(Fig. 3)). Infected control cells exhibited a plaque-forming unit (PFU) titer of 49,600 ± 14,708 PFU/mL, whereas CA-treated cells showed markedly reduced titers: 2,153 ± 1,608 PFU/mL and 67 ± 115 PFU/mL at 2.5 and 5 µM, respectively. Notably, no infectious viral particles were detected at 10 and 20 µM CA concentrations (Figure 3B-C(Fig. 3)).

### Investigation of the potential mechanisms of CA by RNA-seq

#### DEGs following CA treatment

**Figure 4:**
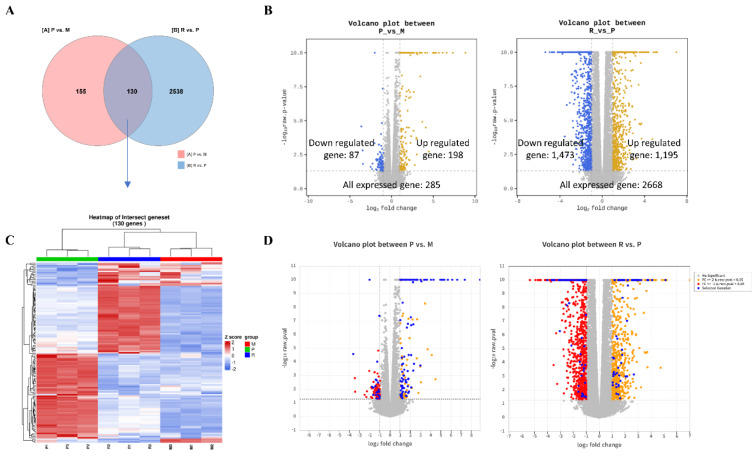
Chlorophyll *a* (CA) modulates antiviral gene expression in vitro, as determined by RNA sequencing. RNA-seq analysis was conducted using Vero E6 cells from three experimental groups: non-treated, non-infected cells (Mock, M; *n* = 3), ZIKV-infected cells (positive control, P; *n* = 3), and CA-treated, ZIKV-infected cells (R; *n* = 3). (A) Venn diagram illustrating the overlap of gene sets from M vs. P and P vs. R. (B) Volcano plot of all expressed genes. (C) Heatmap of 130 differentially expressed genes (DEGs) from the intersecting sets. (D) Volcano plot highlighting the 130 DEGs

To elucidate the molecular mechanisms underlying CA's antiviral activity, transcriptomic analyses were performed on Vero E6 cells under three conditions: untreated, uninfected control (Mock; M); ZIKV-infected (positive control; P); and CA-treated, ZIKV-infected (R). DEGs were identified using criteria of absolute fold change ≥ 2 and raw *p* < 0.05 in at least one comparison (Supplementary information, Figure S3), yielding 2,823 DEGs. Of them, 130 DEGs were commonly altered across both comparisons (Figure 4A(Fig. 4) and Table S1). In the P group relative to M, 87 genes were downregulated and 198 were upregulated. In the R group relative to P, 1,473 genes were downregulated and 1,195 were upregulated (Figure 4B(Fig. 4)). Hierarchical clustering of the 130 shared DEGs revealed four distinct gene expression clusters (Figure 4C(Fig. 4) and Figure S4). Notably, two of these clusters showed similar expression patterns between M and R, whereas all clusters differed significantly between M and P. Genes that were upregulated in P but downregulated in R relative to P included those involved in interferon-inducible antiviral responses (Supplementary information, Figure S4 and Table S2), indicating that CA treatment suppresses the pathological gene expression signature induced by ZIKV infection. These 130 shared DEGs are represented as blue dots in the volcano plot (Figure 4D(Fig. 4)).

#### Enrichment analysis (GO analysis)

**Figure 5:**
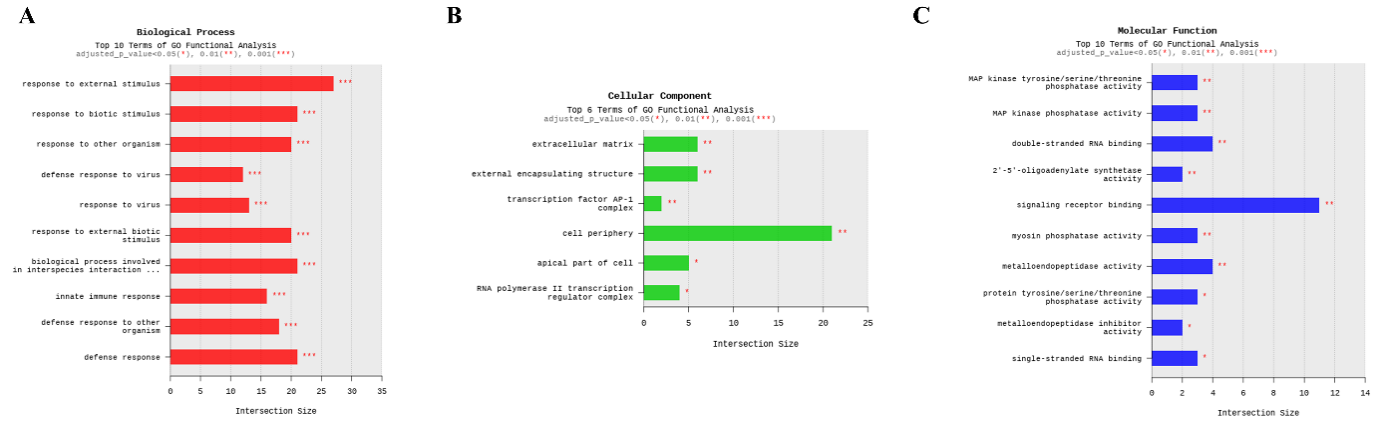
Enrichment analysis of Gene Ontology (GO) terms among the 130 DEGs. Bar plots represent enriched terms related to (A) Biological Process, (B) Cellular Component, and (C) Molecular Function. The x-axis indicates the number of DEGs associated with each GO term (intersection size), and the y-axis lists the corresponding terms

**Figure 6:**
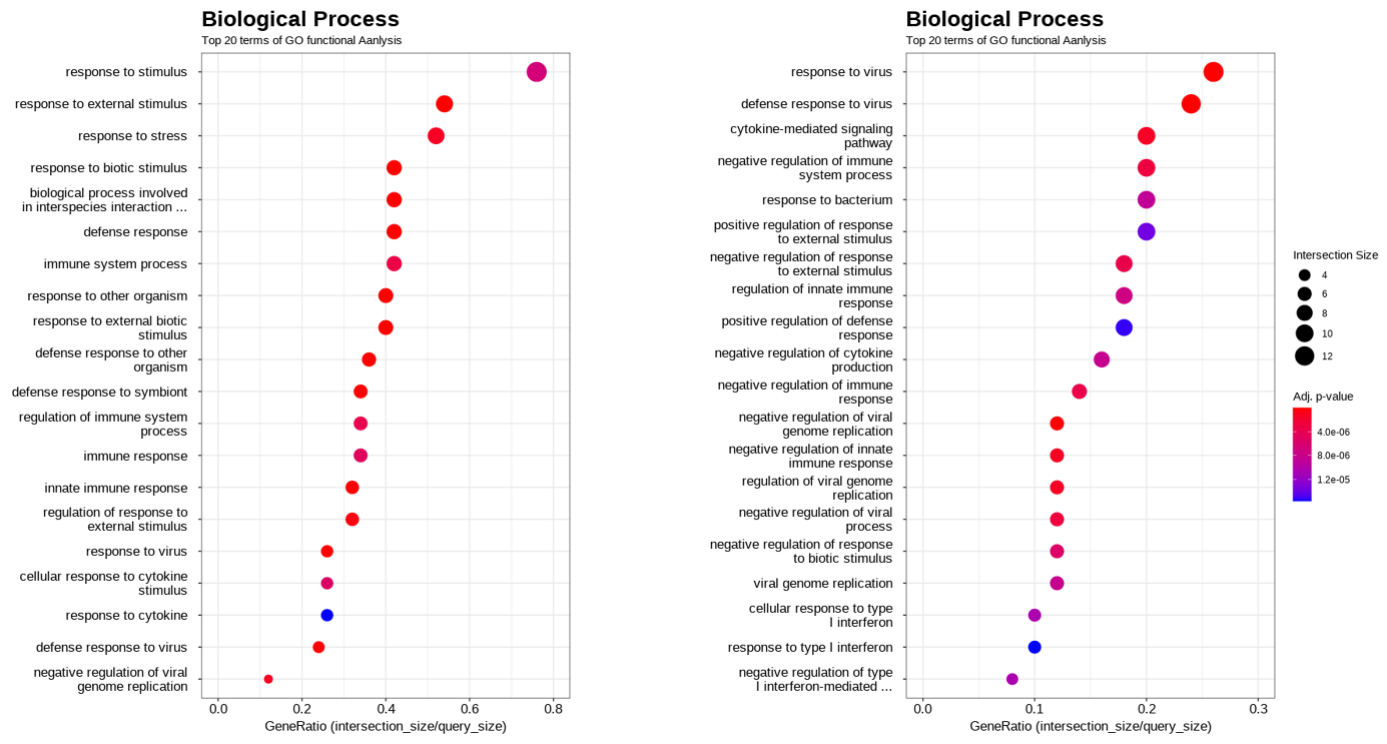
Dot plot of significantly enriched GO Biological Process (BP) terms among the 130 DEGs. The x-axis shows the gene ratio (number of DEGs associated with a GO term divided by the total number of DEGs). Dot size represents the number of DEGs, and color intensity corresponds to statistical significance (-log₁₀ adjusted *p*-value)

To further explore the functional implications of DEGs, all DEGs were categorized into three GO domains, biological process (BP), molecular function, and cellular component, based on annotations from the GO database (Figure 5-6(Fig. 5)(Fig. 6) and Supplementary information, Figure S5-S9). Figure 5(Fig. 5) presents the top enriched GO terms in each category derived from the functional annotation of DEGs. Among these categories, BP terms exhibited the strongest DEG associations, as indicated by intersection size. The most enriched BP terms included “response to external stimulus,” “response to biotic stimulus,” “biological process involved in interspecies interaction,” and “defense response,” suggesting a prominent role for immune-related and interspecies interaction pathways. Figure 6(Fig. 6) shows the 20 most significantly enriched BP terms, ranked by gene ratio and adjusted *p*-value using dot plot visualization. The gene ratio, defined as the proportion of DEGs associated with a given GO term relative to the total number of DEGs, was used as an enrichment metric. The top-ranked terms, including “response to stimulus,” “response to external stimulus,” “response to stress,” and “response to biotic stimulus,” further support the involvement of stress- and stimulus-related pathways. Collectively, these findings indicate that “response to external stimulus” and “response to biotic stimulus” are key biological processes modulated by CA treatment in virus-infected cells. 

#### Key molecules mediating the antiviral activity of CA

**Figure 7:**
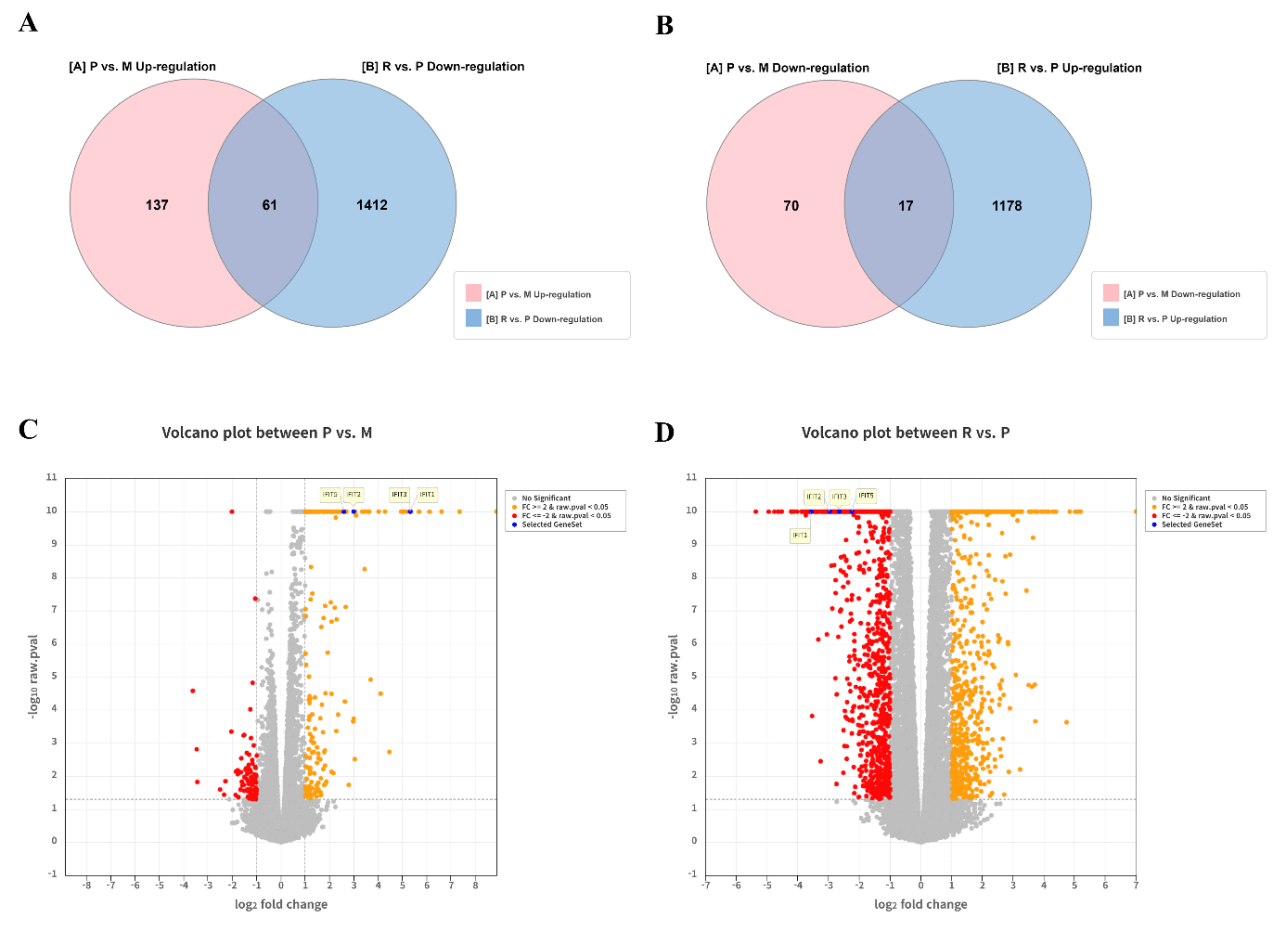
Venn diagrams and volcano plots illustrating differentially expressed genes (DEGs) modulated by CA treatment following ZIKV infection. (A) Venn diagram of downregulated and (B) upregulated DEGs. (C) Volcano plot of downregulated and (D) upregulated DEGs. Experimental groups include non-treated, non-infected cells (Mock, M; *n* = 3), ZIKV-infected cells (positive control, P; *n* = 3), and CA-treated, ZIKV-infected cells (R; *n* = 3)

To identify key genes contributing to the antiviral activity of CA, Venn diagram analysis was performed using DEGs that displayed contrasting expression trends between two pairwise comparisons (Figure 7(Fig. 7)). Genes showing restored expression after CA treatment, following virus-induced dysregulation, were considered candidates. Among the genes upregulated in ZIKV-infected cells (P) relative to the mock group (M), 61 were downregulated upon CA treatment (R) compared to P (Figure 7A(Fig. 7) and Table S3). Conversely, 17 genes that were downregulated in P relative to M were upregulated in R relative to P (Figure 7B(Fig. 7) and Table S4). These results suggest that CA modulates the expression of genes dysregulated by ZIKV infection, thereby contributing to its antiviral efficacy. Notably, the recovered genes included interferon-related genes such as interferon-induced protein with tetratricopeptide repeats (IFIT)1, IFIT2, IFIT3, and IFIT5 (Figure 7C-D(Fig. 7), Table 2(Tab. 2)). 

### qPCR and immunocytochemistry validation of selected DEGs

**Figure 8:**
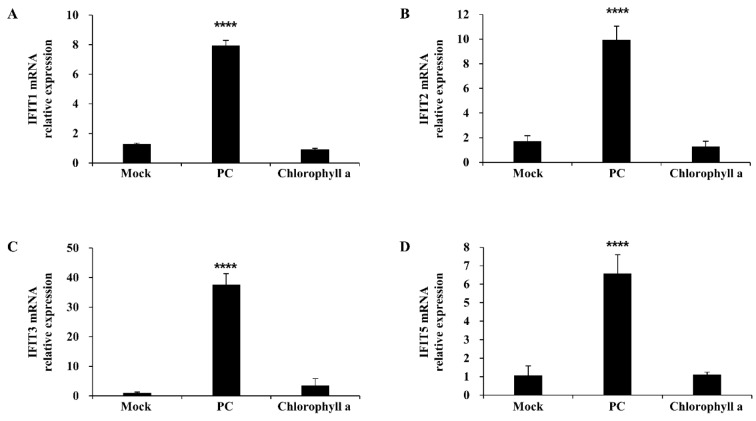
Effect of CA on the relative mRNA expression levels of interferon-stimulated genes. qPCR analysis of (A) *IFIT1*, (B) *IFIT2*, (C) *IFIT3*, and (D) *IFIT5*

**Figure 9:**
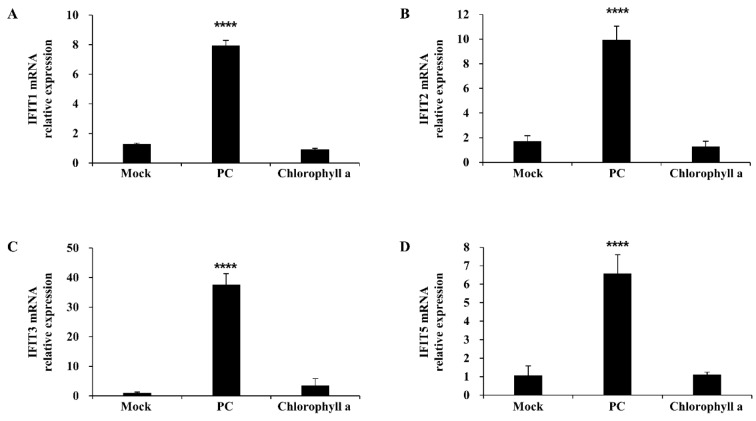
Effect of CA on the protein expression level of IFIT2

**Figure 10:**
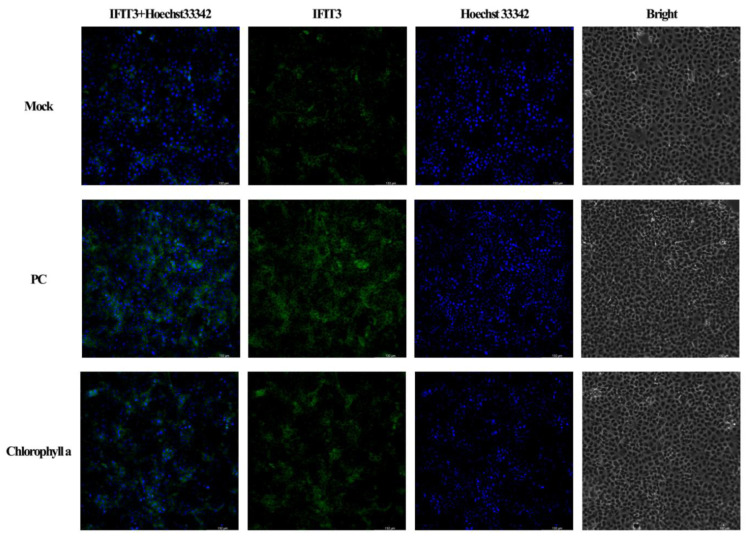
Effect of CA on the protein expression level of IFIT3

To validate the RNA-seq results, the expression levels of *IFIT1*, *IFIT2*, *IFIT3*, and *IFIT5* mRNA were assessed by qPCR following CA treatment (Figure 8(Fig. 8)). ZIKV infection markedly upregulated mRNA expression of all four IFITs relative to the levels observed in the untreated, uninfected control group (M). In contrast, CA treatment restored IFIT expression to levels comparable to those in M (Figure 8(Fig. 8)). Also, the protein expression levels of *IFIT2 *and *IFIT3* were assessed by immunocytochemistry following CA treatment (Figure 9-10(Fig. 9)(Fig. 10), and Figure S10). ZIKV infection markedly upregulated *IFIT2 *and *IFIT3* protein expression relative to the levels observed in the untreated, uninfected control group (M). In contrast, CA treatment reduced *IFIT2 *protein (Figure 9(Fig. 9), and Figure S10a) and *IFIT3 *protein (Figure 10(Fig. 10), and Figure S10b) expression. These findings indicate that CA exerts antiviral effects against ZIKV infection by modulating the expression of interferon-inducible antiviral effectors, particularly IFIT family members.

The search for antiviral agents from natural sources has intensified in recent years. For instance, oridonin, a diterpenoid compound isolated from *Rabdosia rubescens*, has been shown to mitigate herpes simplex virus infection (Jiang et al., 2022[12]). Similarly, several flavonoids (baicalein, fisetin, and quercetagetin) exhibit inhibitory activity against chikungunya virus, a mosquito-borne alphavirus (Lani et al., 2016[15]). Lignans and phenylpropanoids derived from the fruits of *Illicium verum*, a traditional spice, showed antiviral activities against influenza virus A (Li et al., 2022[19]). Extracts and/or compounds from flowering plants such as *Lippia alba*, *Morus* spp., *Tecoma* sp., and *Cryptolepis* sp. also display antiviral properties against ZIKV, coronaviruses, and tobacco mosaic virus (Reis et al., 2020[24]; Sobrinho et al., 2021[28]; Thabti et al., 2020[31]; Zhou et al., 2025[46]). Despite their potential, the limited biomass availability of terrestrial plants often hampers their industrial applicability. In contrast, *Tetraselmis* sp., a marine microalga, is amenable to large-scale cultivation, suggesting that CA derived from *Tetraselmis* sp. represents a viable candidate for commercial antiviral ingredient development. 

RNA-seq, a high-throughput next-generation sequencing technique, enables comprehensive profiling of transcriptomes and has transformed transcriptomics by allowing multidimensional analysis of gene expression at scale (Chen et al., 2016[5]; Yang et al., 2018[39]; Zhang et al., 2021[45]). Also, GO analysis is a widely used tool for elucidating the biological functions underlying gene expression patterns (Rosati et al., 2024[26]). In this study, we investigated the underlying mechanisms of CA by analyzing gene expression profiles in ZIKV-infected cells with or without CA treatment via RNA-seq. The results indicate that “response to external stimulus” and “response to biotic stimulus” are key biological processes modulated by CA treatment in virus-infected cells. Interferons (IFNs) are a group of signaling proteins secreted by cells in response to diverse stimuli, particularly viral infections (Deng et al., 2024[7]; Swaraj and Tripathi, 2024[30]; Wu, 2019[34]). For this reason, IFNs are commonly used as drugs to treat viral infection (Zhang et al., 2016[43]). IFN signaling induces the expression of interferon-stimulated genes (ISGs), which act as downstream effectors to restrict viral replication and modulate immune responses. Proteins encoded by ISGs inhibit viral propagation by suppressing viral transcription, translation, and replication. In addition, ISGs contribute to the degradation of viral nucleic acids and reprogram host lipid metabolism to counteract viral infection (Li et al., 2022[17]). The IFIT gene family, classified as ISGs, is a crucial component of the antiviral innate immune response. These proteins inhibit various stages of viral replication, including translation initiation, viral propagation, and protein synthesis (Wu et al., 2023[36]; Xu et al., 2022[38]). Four IFIT family members have been identified in humans, distinguished by the number of tetratricopeptide repeats they contain (Vladimer and Górna, 2014[33]). Our findings, supported by RNA-seq and qPCR analyses, indicate the involvement of the entire IFIT family in the antiviral mechanism of CA.

In the RNA-seq analysis on the antiviral activity of CA, a limitation of this study is the absence of a control group that exclusively received CA, which hinders the ability to ascertain whether the observed effects were solely attributable to the antiviral action of CA or were also influenced by its direct cellular toxicity or transcriptional modulation. Another limitation is that the experiments were conducted using a single cell line, and it is possible that the results may not fully reflect physiological diversity. To overcome these limitations and more clearly validate the antiviral activity of CA, further studies should include well-structured experimental designs involving multiple cell lines, time-course experiments, and appropriate control groups such as non-treated, non-infected cells (Mock), CA-treated cells (Control), ZIKV-infected cells (PC), CA-treated, ZIKV-infected cells (CA), and vehicle controls.

Antiviral mechanisms are closely associated with the viral replication cycle. Since certain viruses share similarities in their replication processes, some antiviral agents can inhibit multiple viral infections-these are known as broad-spectrum antivirals. Such agents function either by enhancing host antiviral responses or by suppressing host factors essential for viral replication. Thus, these agents are not directly influenced by viral genetic control, and the likelihood of resistance mutations is relatively low. Therefore, the discovery of broad-spectrum antivirals has gained increasing attention in recent years. Notably, several chlorophyll derivatives have been reported to exhibit antiviral activity against dengue virus (Azizullah et al., 2014[1]) and hepatitis C virus (Ratnoglik et al., 2014[23]). In addition, chlorophyll derivatives are known to possess a wide range of biological activities, including anticancer, antimutagenic, antioxidant, radioprotective, antimicrobial, and anti-inflammatory effects. Also, CA is consumable as a dietary component, and it holds potential as a functional food ingredient or nutraceutical supplement for health improvement. In future studies, it will be important to evaluate the antiviral efficacy of CA not only against flaviviruses but also across a broader spectrum of viral species, and to further assess its potential as a food or pharmaceutical material through comprehensive ADMET profiling and long-term safety studies. 

## Conclusion

Overall, in this study, we employed RNA-seq as a systematic approach to investigate the antiviral activity and underlying mechanisms of CA by analyzing gene expression profiles in ZIKV-infected cells with or without CA treatment. A total of 130 DEGs were identified in response to CA. Venn diagram and volcano plot analyses indicated the potential involvement of the entire IFIT family in the antiviral activity of CA. This was further supported by qPCR validation of *IFIT1*, *IFIT2*, *IFIT3*, and *IFIT5* mRNA expression levels and immunocytochemistry validation of *IFIT2* and *IFIT3* protein expression levels. Thus, CA restores the expression of key interferon-stimulated genes and modulates innate antiviral immune pathways suppressed by ZIKV. This means that CA not only inhibits viral replication but also reprograms host cell signaling to enhance antiviral immunity. This mechanistic insight contributes new molecular-level knowledge regarding the antiviral properties of marine microalgal metabolites. 

Microalgae, including *Tetraselmis* sp., represent a promising avenue for developing more sustainable and productive agricultural practices (Yao et al., 2018[41]; Zhang et al., 2025[44]). Recent advances in the large-scale cultivation of *Tetraselmis* sp. in semi-open raceway systems installed in a greenhouse have enabled year-round production of this marine microalga. Moreover, cultivation systems allow for the controlled production of pharmaceutically relevant compounds, including chlorophyll derivatives, through modulation of parameters such as temperature, salinity, and circulation speed (Lee et al., 2021[16]). These agricultural advantages make it possible for CA extracted from *Tetraselmis* sp. to be developed as a sustainable antiviral ingredient for future generations. 

In summary, our findings support the potential industrial application of CA derived from *Tetraselmis* sp. as a promising antiviral candidate against ZIKV infection. This study holds broader implications for the development of sustainable, algae-based antiviral agents and functional food additives capable of mitigating viral infections. Our findings support the agricultural-food-nutrition continuum by demonstrating that marine microalgae can serve as a high-value, renewable resource for developing functional food ingredients and nutraceuticals with molecular-level confirmed antiviral potential. However, continued research and development is essential to unlock their full potential and integrate them effectively into mainstream agriculture.

## Declaration

### Supporting information 

Throughput comparison between raw data and trimmed data; Q30 value comparison between raw data and trimmed data; Heat map for differentially expressed genes (DEGs) identified using criteria of absolute fold change ≥ 2 and raw p < 0.05 in at least one comparison; Heatmap of 130 DEGs from the intersecting sets; Bar plots for enrichment analysis of Gene Ontology (GO) terms related to Biological Process among the 130 DEGs; Bar plots for enrichment analysis of GO terms related to Cellular Component among the 130 DEGs; Dot plot of significantly enriched GO Cellular Component terms among the 130 DEGs; Bar plots for enrichment analysis of GO terms related to Molecular Function among the 130 DEGs; Dot plot of significantly enriched GO Molecular Function terms among the 130 DEGs; Corrected integrated density of the stained cells with IFIT2 (A) and IFIT3 (B) by immunocytochemistry(PDF); List for all identified genes; Heatmap category for 130 differentially expressed genes; List for DEGs upregulated by CA treatment following ZIKV infection; List for DEGs downregulated by CA treatment following ZIKV infection (Excel).

### Acknowledgments

This research was supported by Korea Institute of Marine Science and Technology Promotion (KIMST) funded by the Ministry of Oceans and Fisheries, Korea (20210466).

### Author contributions

The manuscript was written by all authors. All authors have given approval of the final version of the manuscript. Conceptualization, writing-original draft preparation, NK; Methodology, investigation, EAK; Data curation, Formal analysis, YJL; Data curation, Formal analysis, SYH; Software, JHH; Validation, Resources, WKL; Visualization, Resources, YKR; Resources, TK; Writing-review and editing, project administration, supervision, SJH.

### Data availability 

All data in this study can be downloaded from the links in this article. The data supporting the findings of this study are available from the corresponding author upon request.

### Conflicts of interest

The authors declare that they have no conflict of interest.

### Artificial Intelligence (AI) - assisted technology

No artificial intelligence tools were used in the preparation, writing, editing, or analysis of this manuscript.

## Supplementary Material

Supplementary information

Table S1 List for all identified genes

Table S2 Heatmap category for 130 differentially expressed genes

Table S3 List for DEGs upregulated by CA treatment following ZIKV infection

Table S4 List for DEGs downregulated by CA treatment following ZIKV infection

## Figures and Tables

**Table 1:**
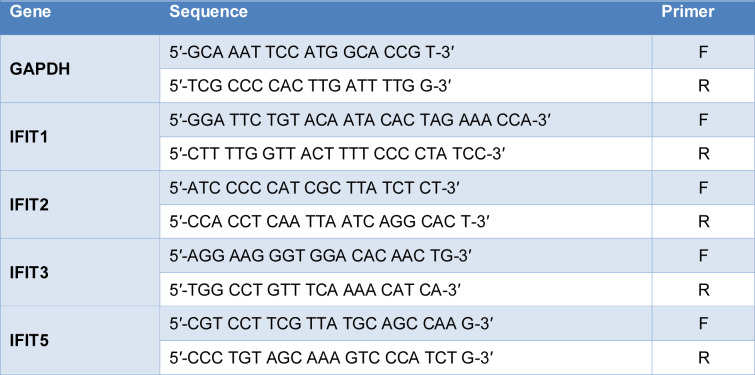
Primer sequences

**Table 2:**
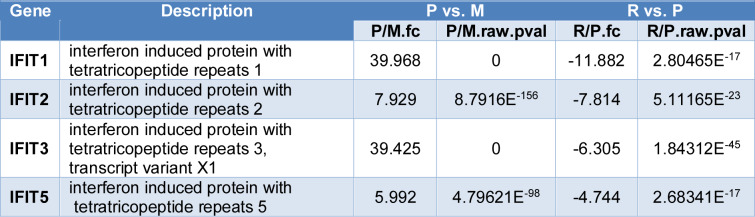
Regulated genes by CA treatment in ZIKV-infected cells

**Figure 1:**
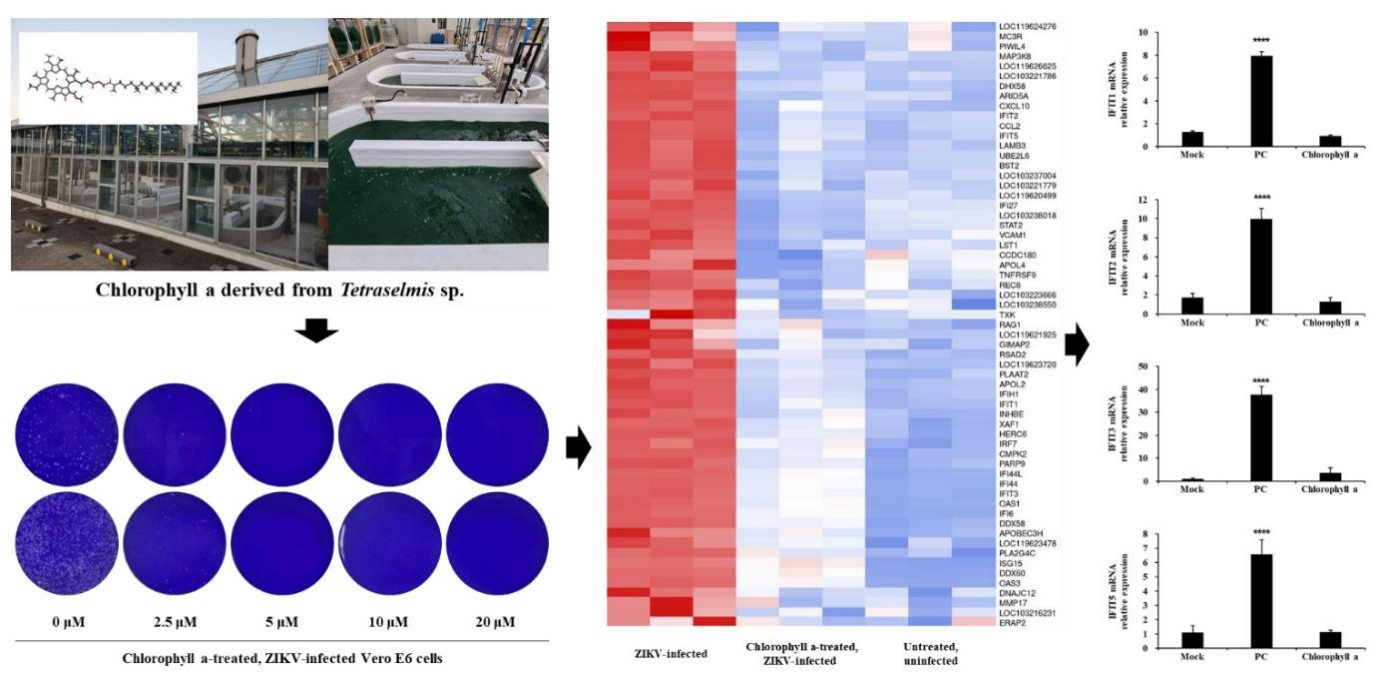
Graphical abstract
