# Supplementary material for: RNA-Seq-based investigation of interferon-mediated antiviral activity of chlorophyll a from Tetraselmis sp
Source: EXCLI J. 2026 Jan 9;25:150–66. doi: 10.17179/excli2025-8841 (PMC12901952; doi:10.17179/excli2025-8841)
Supplement: Supplementary information [file EXCLI-25-150-s-001.pdf]

EXCLI Journal 2026;25:150-166 – ISSN 1611-2156
Received: August 15, 2025, accepted: December 19, 2025, published: January 09, 2026

Supplementary information to:

Original article:

RNA-SEQ-BASED INVESTIGATION OF INTERFERON-MEDIATED ANTIVIRAL ACTIVITY OF CHLOROPHYLL A FROM *TETRASELMIS* SP.

Nalae Kang[1,2], Eun-A Kim[1], Yeon-Ji Lee[1], Seong-Yeong Heo[1,2], Jun-Ho Heo[1], Won-Kyu Lee[1], Yong-Kyun Ryu[1], Taeho Kim[1], Soo-Jin Heo[1,2,*]

[1] Jeju Bio Research Center, Korea Institute of Ocean Science and Technology (KIOST), Jeju 63349, Republic of Korea
[2] Department of Marine Technology & Convergence Engineering, University of Science and Technology (UST), Daejeon 34113, Republic of Korea

* **Corresponding author:** Soo-Jin Heo, Jeju Bio Research Center, Korea Institute of Ocean Science and Technology (KIOST), Jeju 63349, Republic of Korea. Tel.: +82 64 798 6101; E-mail: sjheo@kiost.ac.kr

https://dx.doi.org/10.17179/excli2025-8841

This is an Open Access article distributed under the terms of the Creative Commons Attribution License (https://creativecommons.org/licenses/by/4.0/).

EXCLI Journal 2026;25:150-166 – ISSN 1611-2156
Received: August 15, 2025, accepted: December 19, 2025, published: January 09, 2026

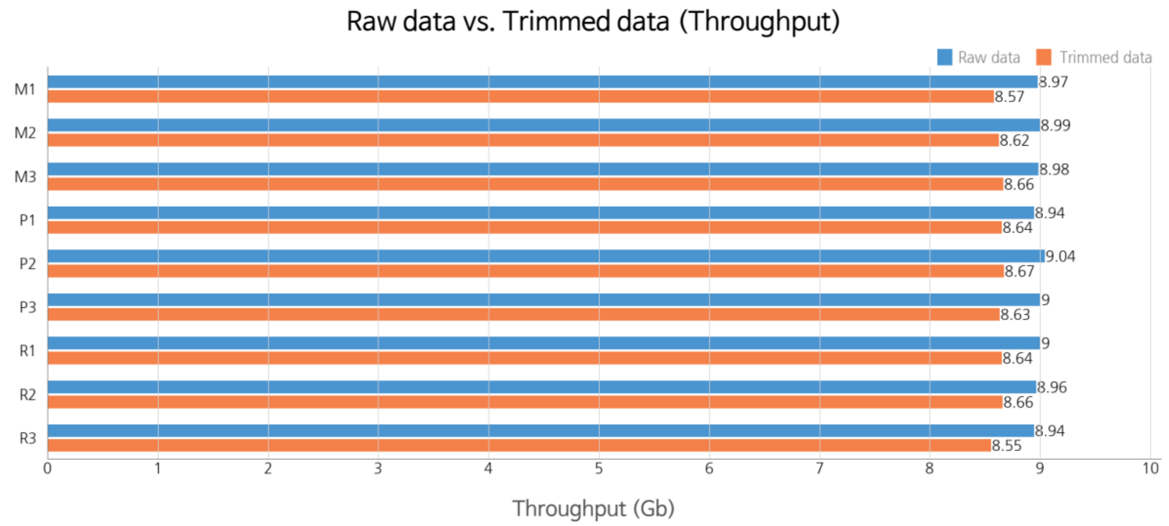

Supplementary Figure 1: Throughput comparison between raw data and trimmed data

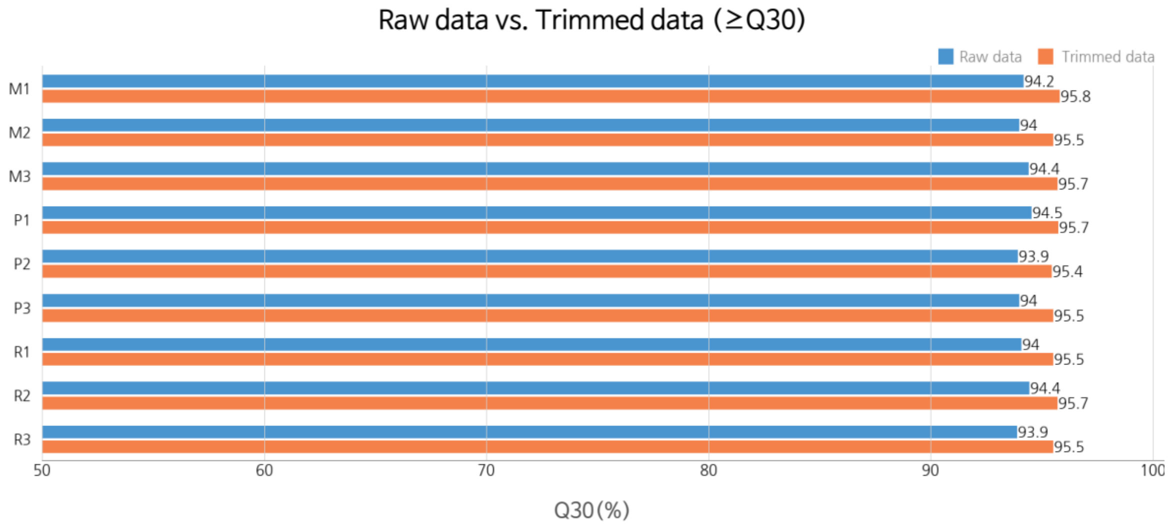

Supplementary Figure 2: Q30 value comparison between raw data and trimmed data

EXCLI Journal 2026;25:150-166 – ISSN 1611-2156
Received: August 15, 2025, accepted: December 19, 2025, published: January 09, 2026

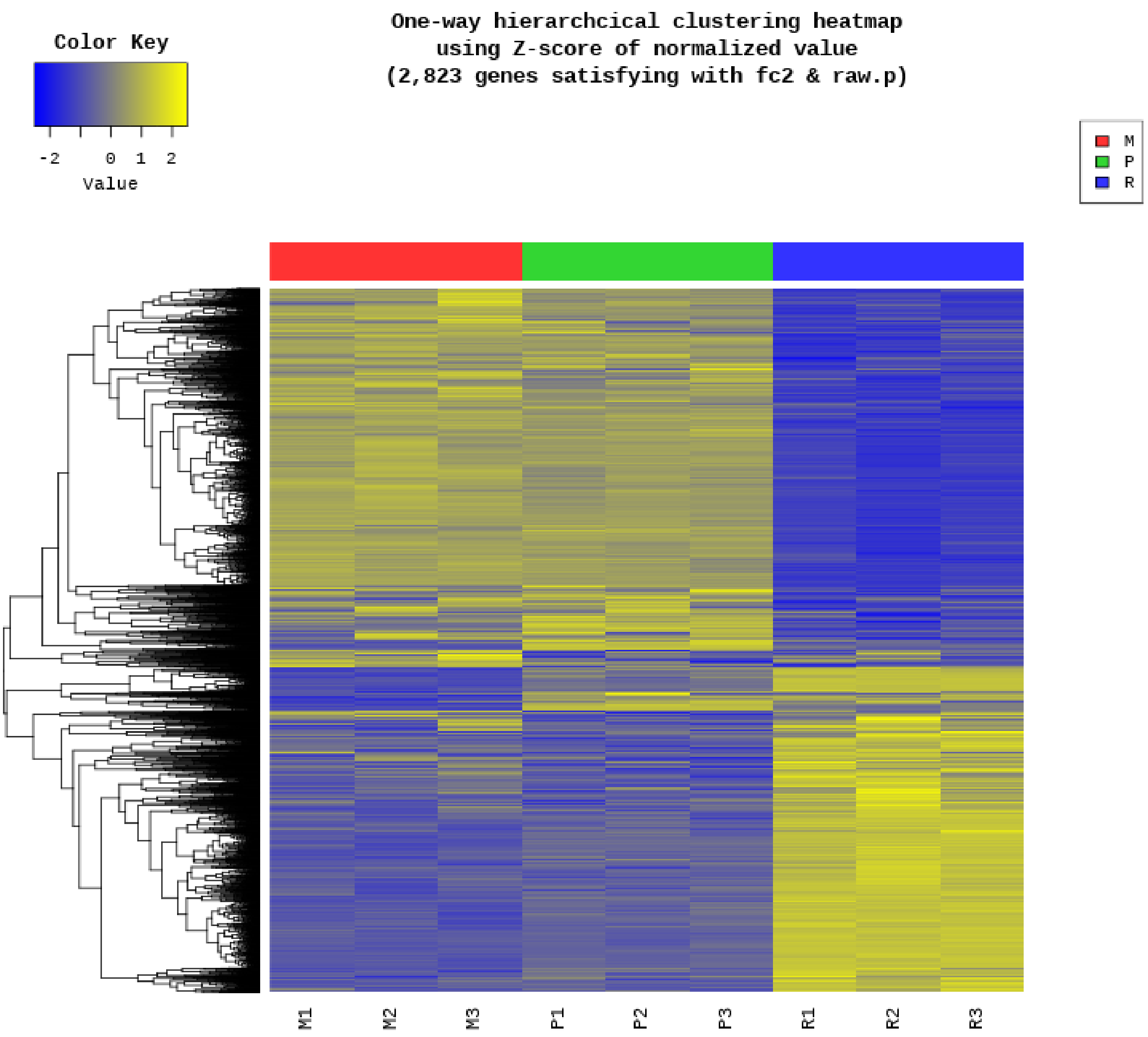

Supplementary Figure 3: Heat map for differentially expressed genes (DEGs) identified using criteria of absolute fold change ≥ 2 and raw $p < 0.05$ in at least one comparison. Untreated, uninfected control (Mock; M); ZIKV-infected (positive control; P); and CA-treated, ZIKV-infected (R)

EXCLI Journal 2026;25:150-166 – ISSN 1611-2156
Received: August 15, 2025, accepted: December 19, 2025, published: January 09, 2026

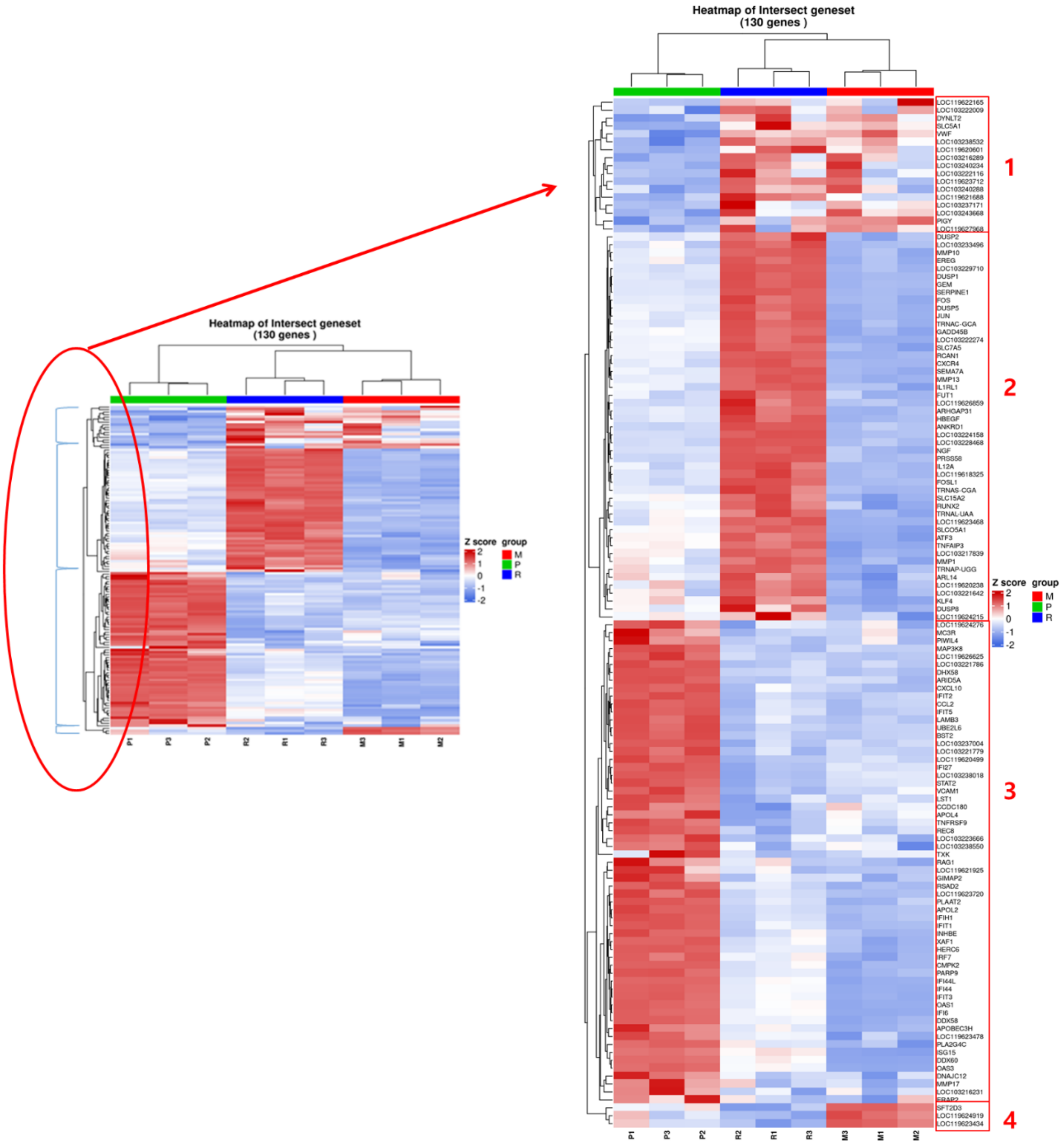

Supplementary Figure 4: Heatmap of 130 DEGs from the intersecting sets

EXCLI Journal 2026;25:150-166 – ISSN 1611-2156
Received: August 15, 2025, accepted: December 19, 2025, published: January 09, 2026

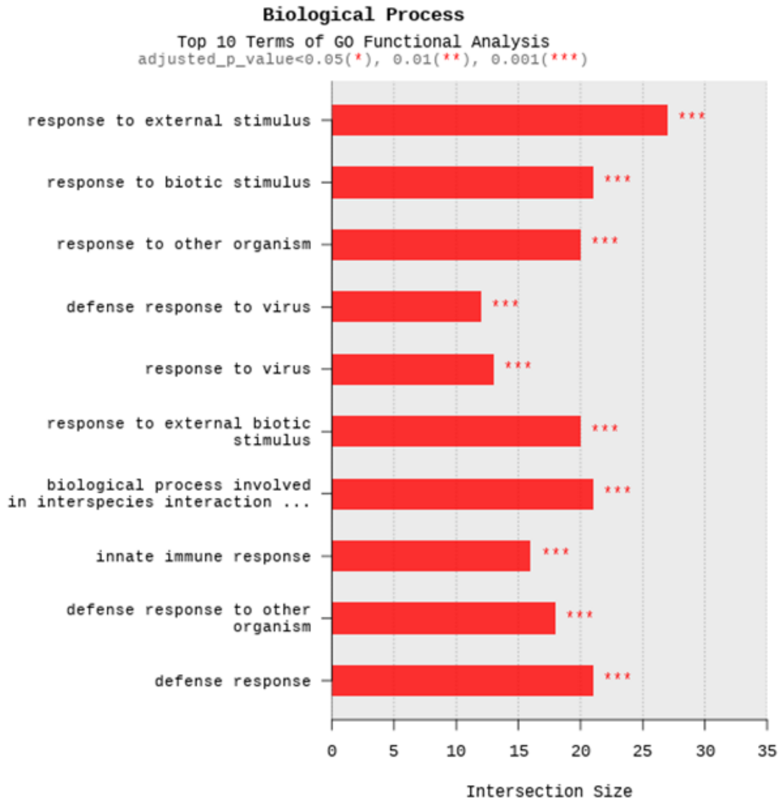

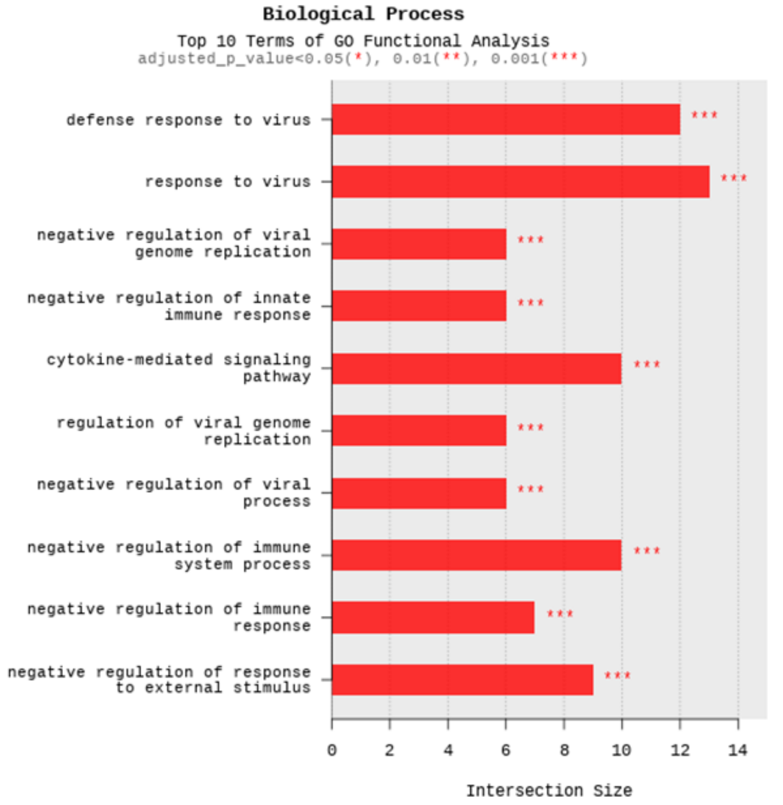

Supplementary Figure 5: Bar plots for enrichment analysis of Gene Ontology (GO) terms related to Biological Process among the 130 DEGs. The x-axis indicates the number of DEGs associated with each GO term (intersection size), and the y-axis lists the corresponding terms.

EXCLI Journal 2026;25:150-166 – ISSN 1611-2156
Received: August 15, 2025, accepted: December 19, 2025, published: January 09, 2026

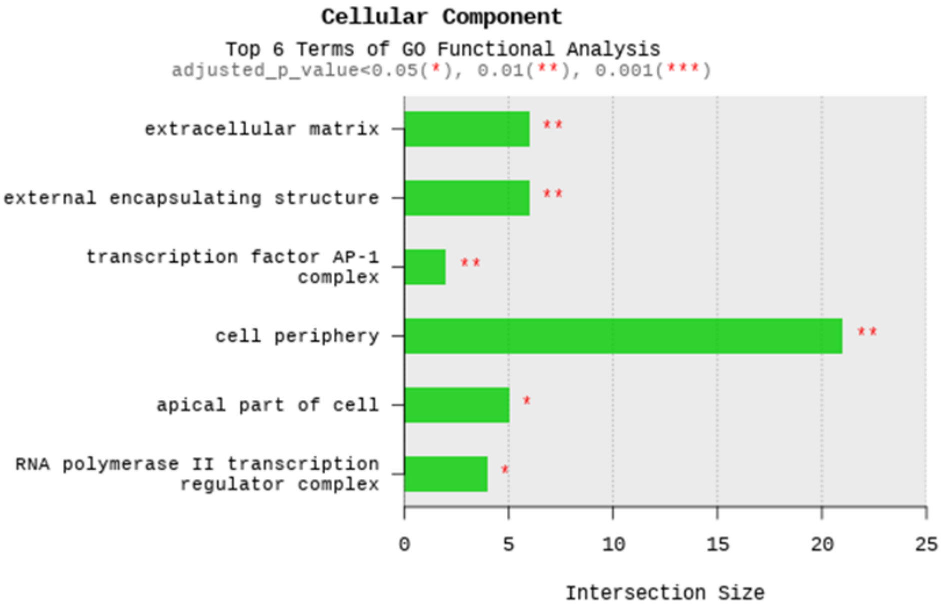

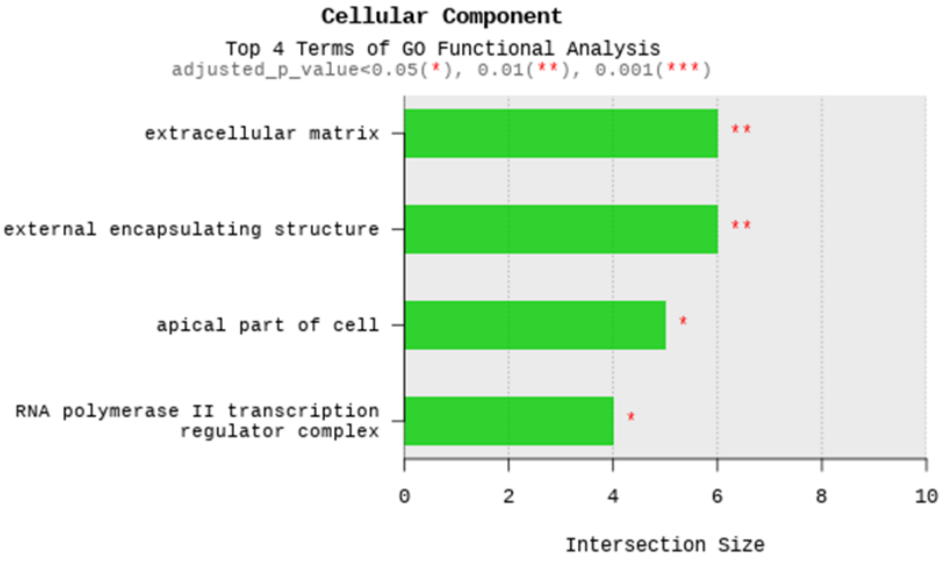

Supplementary Figure 6: Bar plots for enrichment analysis of GO terms related to Cellular Component among the 130 DEGs. The x-axis indicates the number of DEGs associated with each GO term (intersection size), and the y-axis lists the corresponding terms.

EXCLI Journal 2026;25:150-166 – ISSN 1611-2156
Received: August 15, 2025, accepted: December 19, 2025, published: January 09, 2026

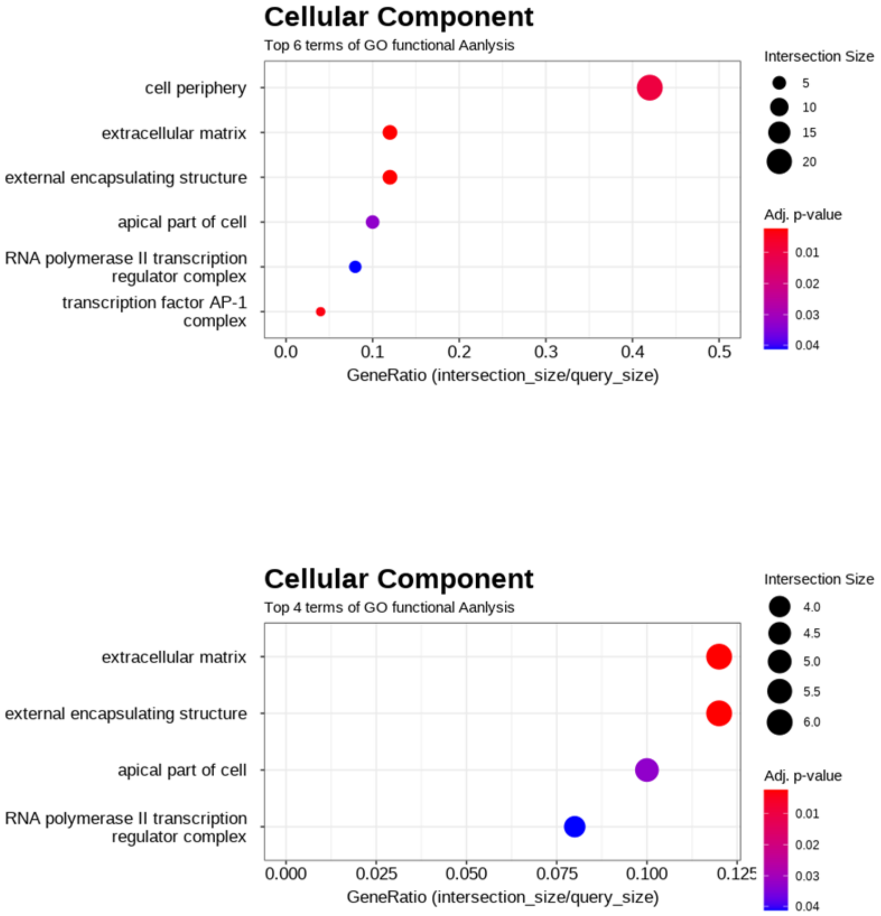

Supplementary Figure 7: Dot plot of significantly enriched GO Cellular Component terms among the 130 DEGs. The x-axis shows the gene ratio (number of DEGs associated with a GO term divided by the total number of DEGs). Dot size represents the number of DEGs, and color intensity corresponds to statistical significance (–$\log_{10}$ adjusted p-value).

EXCLI Journal 2026;25:150-166 – ISSN 1611-2156
Received: August 15, 2025, accepted: December 19, 2025, published: January 09, 2026

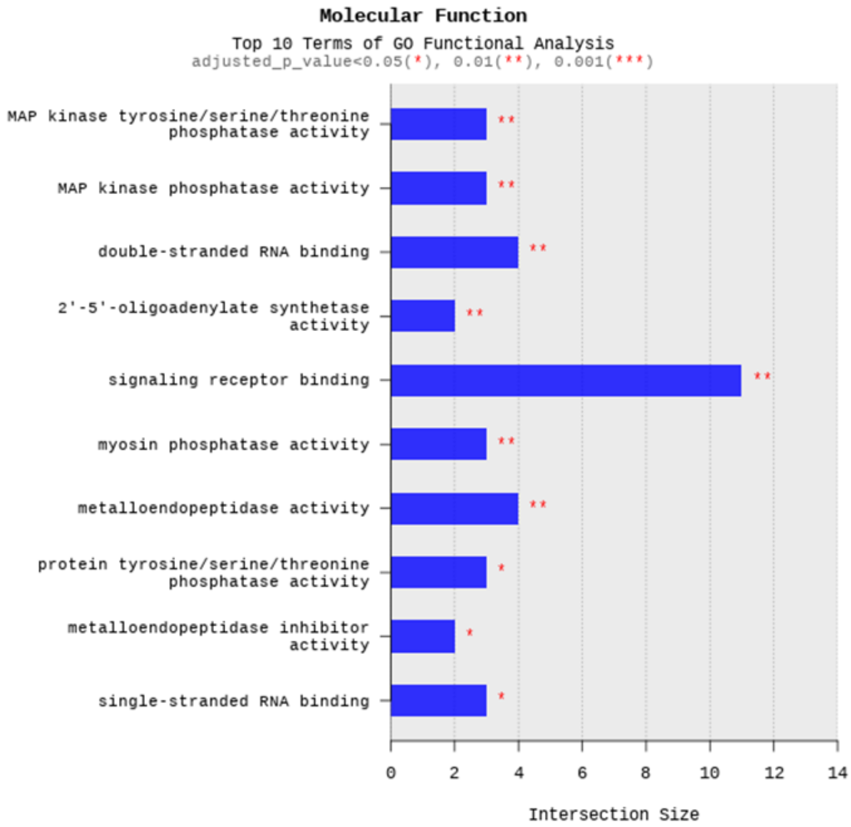

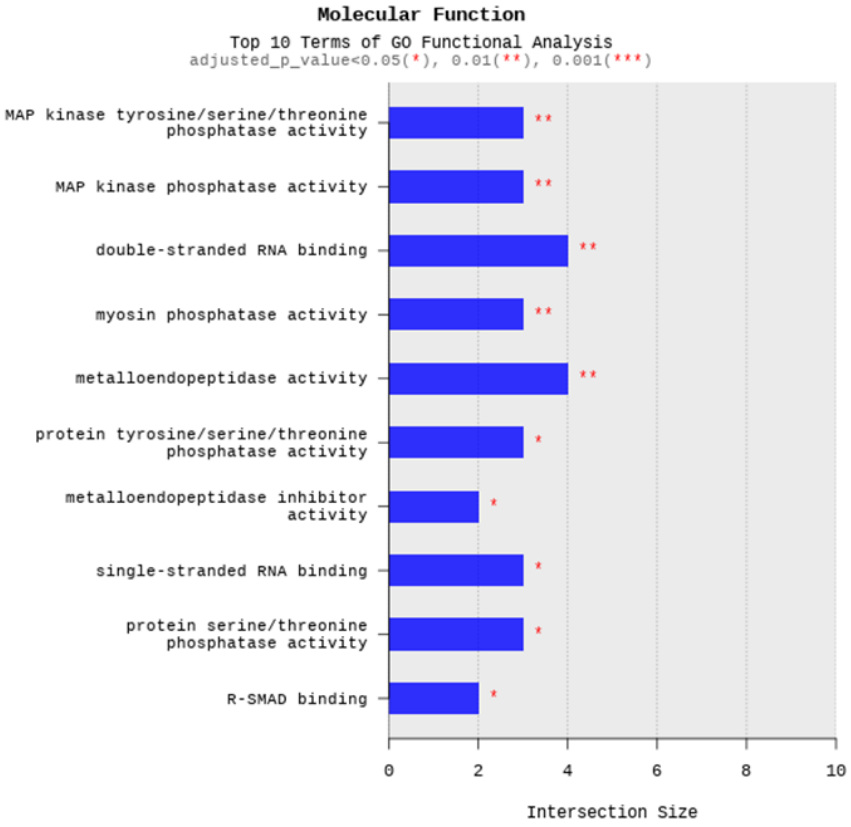

Supplementary Figure 8: Bar plots for enrichment analysis of GO terms related to Molecular Function among the 130 DEGs. The x-axis indicates the number of DEGs associated with each GO term (intersection size), and the y-axis lists the corresponding terms.

EXCLI Journal 2026;25:150-166 – ISSN 1611-2156
Received: August 15, 2025, accepted: December 19, 2025, published: January 09, 2026

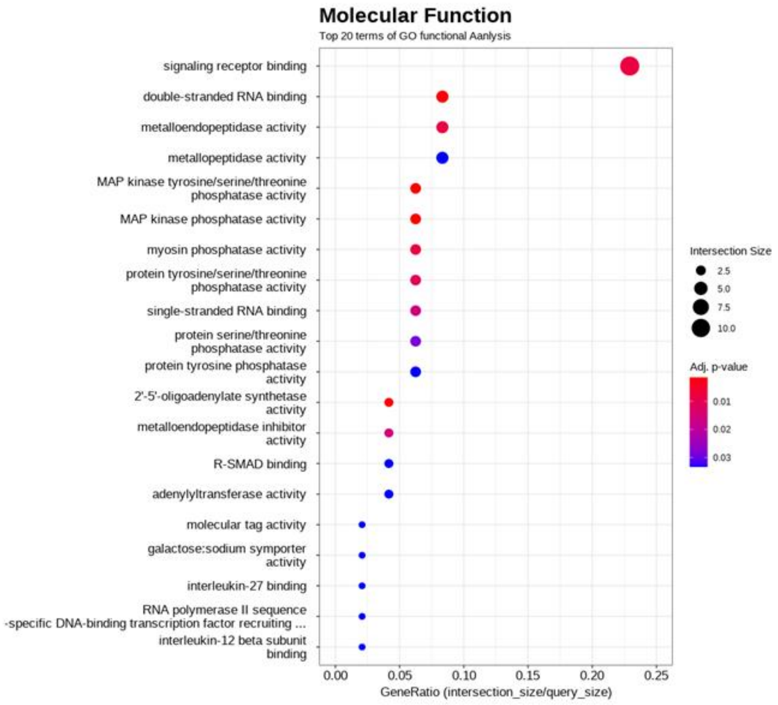

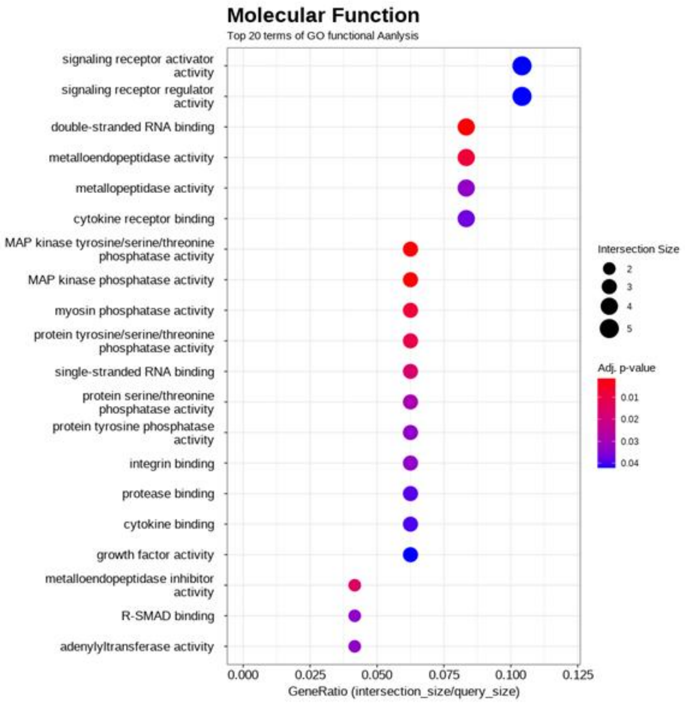

Supplementary Figure 9: Dot plot of significantly enriched GO Molecular Function terms among the 130 DEGs. The x-axis shows the gene ratio (number of DEGs associated with a GO term divided by the total number of DEGs). Dot size represents the number of DEGs, and color intensity corresponds to statistical significance (–$\log_{10}$ adjusted p-value).

EXCLI Journal 2026;25:150-166 – ISSN 1611-2156
Received: August 15, 2025, accepted: December 19, 2025, published: January 09, 2026

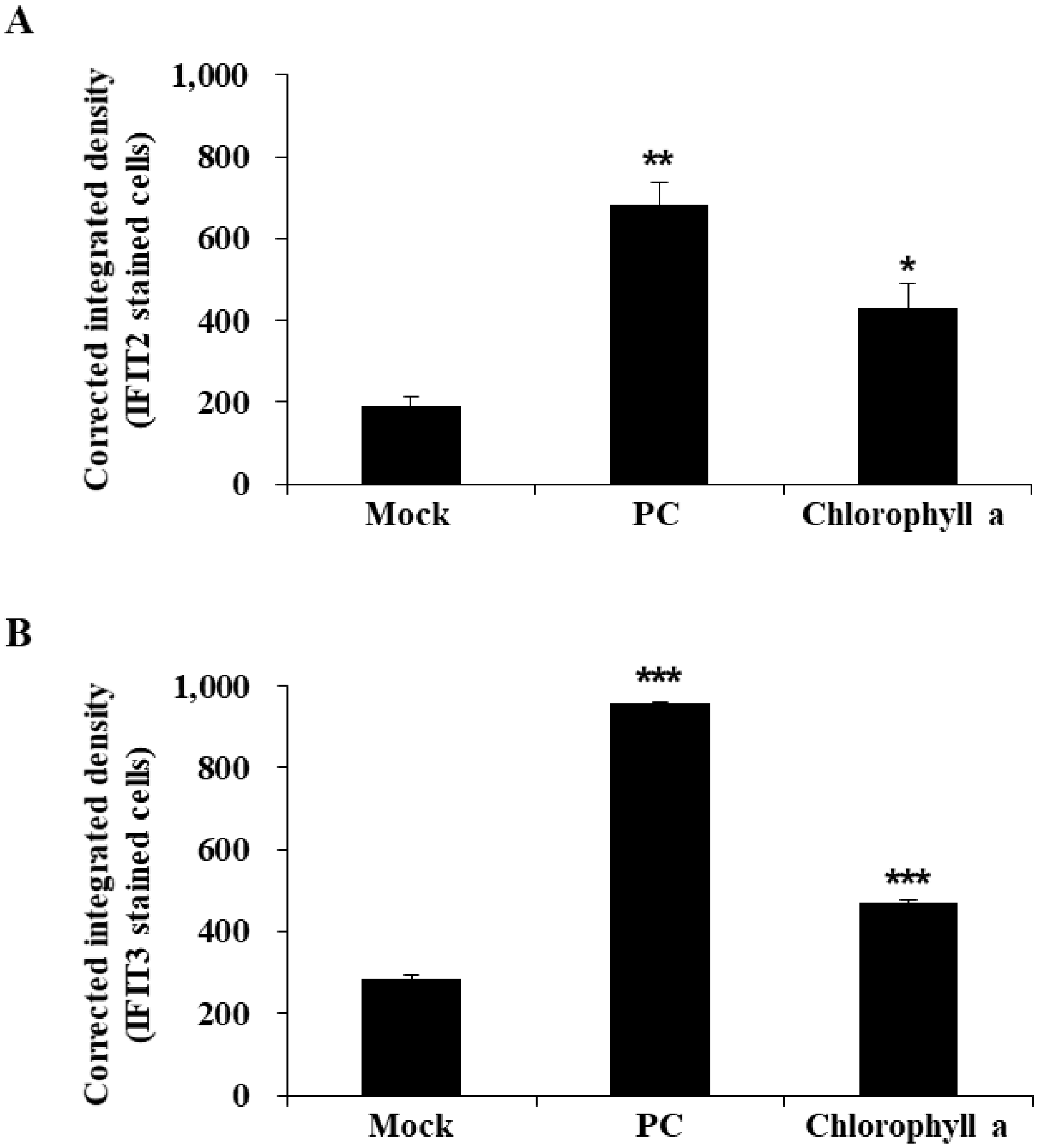

Supplementary Figure 10: Corrected integrated density of the stained cells with IFIT2 (**A**) and IFIT3 (**B**) by immunocytochemistry
